# Microglial/macrophage activation in the cerebrospinal fluid of neuromyelitis optica spectrum disorders

**DOI:** 10.1002/brb3.2798

**Published:** 2022-10-28

**Authors:** Jinghong Li, Yan He, Honghao Wang, Jinyu Chen

**Affiliations:** ^1^ Department of Neurology The First Affiliated Hospital of Zhengzhou University Chenzhou China; ^2^ Department of Neurology Chenzhou No. 1 People's Hospital Chenzhou China; ^3^ Neuroimmunology & Neuroinfection Group, Department of Neurology Nanfang Hospital, Southern Medical University Guangzhou China

**Keywords:** macrophage, microglia, neuromyelitis optica spectrum disorders, sCD14, sCD14, YKL‐40

## Abstract

**Aim:**

The aims of this pilot study were to investigate the levels of biomarkers of microglial/macrophage activation—YKL‐40, sCD163, and sCD14—in patients with neuromyelitis optica spectrum disorder (NMOSD) and determine the possible associations between these biomarkers and Expanded Disability Status Scale (EDSS) scores.

**Methods:**

We measured the levels of three microglia‐/macrophage‐related proteins (YKL‐40, soluble CD163, and soluble CD14) in cerebrospinal fluid (CSF) using enzyme‐linked immunosorbent assays. In addition, patients’ neurological disability levels were assessed using EDSS scores.

**Results:**

NMOSD patients had significantly higher CSF levels of YKL‐40(210.52 ± 161.62 for NMOSD and 63.18 ± 9.22 for control), sCD163 (87.23 ± 56.85 for NMOSD and 58.14 ± 7.66 for control), and sCD14 (68.22 ± 24.11 for NMOSD and 55.75 ± 9.48 for control) compared with controls. Furthermore, these biomarker levels were positively correlated with EDSS scores in patients with NMOSD (*r* = 0.303, *p* = .002 for YKL‐40; *r* = 0310, *p* = .001 for sCD14; *r* = 0.250, *p* = .011 for sCD163), but not in patients with multiple sclerosis or glial fibrillary acidic protein astrocytopathy.

**Conclusion:**

Our findings suggest that microglial/macrophage activation may be implicated in the pathogenesis of NMOSD.

## Graphical abstract

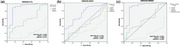

## 1 INTRODUCTION

Neuromyelitis optica spectrum disorder (NMOSD) is a chronic disabling idiopathic inflammatory demyelinating disease of the central nervous system (CNS) and is considered an optic‐spinal form of multiple sclerosis (MS). Until 2004, a specific antibody (NMOSD‐IgG) was suggested to be a biomarker of NMOSD, distinguishing it from conventional MS (Lennon et al., 2004; Wingerchuk et al., 1999). However, the pathogenesis of NMOSD is not completely understood. It is believed that antibodies produced after the activation of B lymphocytes, as well as the involvement of T cells and a large number of complements, play vital roles in the disease. Recently, microglial/macrophage activity has also been described in NMOSD (Kawachi & Lassmann, 2017; Krumbholz et al., 2006).

Microglia belong to the monocyte/macrophage line, which are an important type of resident CNS macrophages cells. They mediate a variety of immune responses in the CNS and interact with peripheral immune cells (Ransohoff & Brown, 2012). Microglia are the most abundant cell type in the human CNS and become important inflammatory mediators when activated, which can lead to marked neuronal damage. Generally, activated microglia cause M1/M2 polarization. In the early stage of inflammation, microglia differentiate into the M1 phenotype and release proinflammatory cytokines, leading to inflammatory damage. In the later stage, microglia transform into the M2 phenotype and participate in the tissue repair process of inflammation (Jespersen et al., 2016; Litvack & Palaniyar, 2010). Recent animal experiments have reported that chemokines expressed by activated microglia can direct leukocytes to the axons of the CNS and cause damage (Liu et al., 2019). Accordingly, neuroimmunity associated with microglia/macrophages appears to be strongly associated with neuroinflammatory diseases.

Some biomarkers directly reflect the process of microglial/macrophage activation (Pranzatelli et al., 2017). Chitinase‐3‐like‐1, also known as YKL‐40, is secreted by activated macrophages and microglia (Craig‐Schapiro et al., 2010; Litvack & Palaniyar, 2010; Qi et al., 2020). Increased levels of YKL‐40 in the cerebrospinal fluid (CSF) are associated with a variety of neuroimmune and neuroinflammatory diseases, such as MS, autoimmune encephalitis, and stroke (Chen et al., 2018; Correale & Fiol, 2011; Hjalmarsson et al., 2014; Oldoni et al., 2020; Qi et al., 2020). CD14, a leukocyte differentiation antigen of activated macrophages/microglia and neutrophils, is a lipopolysaccharide receptor and includes a cell membrane type (mCD14) and a soluble type (sCD14) (Jespersen et al., 2016). Both protein and mRNA expression of sCD14 have been detected in microglia, suggesting that microglia may be the cellular source of sCD14 in the CSF. In addition, sCD14 can moderately enhance the phagocytic activity of microglia (Litvack & Palaniyar, 2010). CD163 is expressed in activated microglia and in perivascular and meningeal macrophages (Stilund et al., 2014; Zhang et al., 2011). During the inflammatory state, membrane‐bound CD163 detaches from the cell surface and becomes soluble (sCD163). sCD163, a microglial biomarker, has recently been reported to play a role in downstream anti‐inflammatory and antioxidant responses by inhibiting the proliferation and activation of T lymphocytes (Hasegawa et al., 2013). We therefore investigated these three markers in the CNS as potential biomarkers of microglial/macrophage activation.

Evidence is accumulating for microglial/macrophage activation in patients with MS, some CNS infections, and brain aging and injury (Hasegawa et al., 2013; Kamat et al., 2012; Møller, 2012; Lutterotti et al., 2006; Parkner et al., 2012). However, little is known about microglial/macrophage activation in NMOSD. In the present study, we aimed to determine the levels of YKL‐40, sCD163, and sCD14 in the CSF of patients with NMOSD, and to evaluate the association between these markers and Expanded Disability Status Scale (EDSS) scores.

## 2 MATERIALS AND METHODS

### 2.1 Patients and controls

In this study, we included 103 first diagnosis or patients with relapsing NMOSD consistent with the 2015 Wingerchuk diagnostic criteria (Wingerchuk et al., 2015), 15 patients with relapsing–remitting MS (RRMS) fulfilling the 2017 McDonald's diagnostic criteria (Thompson et al., 2018), 20 patients with autoimmune glial fibrillary acidic protein (GFAP) astrocytopathy meeting the 2018 diagnostic criteria (Fang et al., 2016), and 16 controls with noninflammatory neurological diseases included: (a) seven patients with cervical spondylopathy, (b) five patients with lumbar spondylopathy, and (c) four patients with migraine. All subjects were recruited from the Department of Neurology, Nanfang Hospital, Southern Medical University. Clinical relapse in patients with MS and NMOSD was defined as the onset of new symptoms that lasted at least 24 h, with an increase of more than 1.0 in the EDSS compared with on admission. All enrolled patients were diagnosed by two neurologists, and all samples were collected before treatment. The clinical and demographic characteristics of the patients are shown in Table 1. Exclusion criteria included other organ co‐infections; having received any other investigational drug within 3 months prior to baseline. All patients provided written informed consent prior to randomization.

**TABLE 1:** Demographic and clinical features of the patients and controls

	NMOSD (*n* = 103)	RRMS (*n* = 15)	GFAP (*n* = 20)	CTLs (*n* = 16)
Gender (male/female)	13/90	6/9	13/7	11/5
Age (years)	43.99 ± 15.76	35.53 ± 11.56	38.70 ± 11.50	33.90 ± 2.00
Clinical symptoms Fever Dizziness Disorders of behavior or cognition Eye pain or vision loss Abnormal feeling Autonomic disturbances Abnormal movements Epilepsy Vomit	8 (8%) 17 (17%) 1 (1%) 33 (32%) 56 (54%) 45 (44%) 1 (1%) 7 (7%) 18 (17%)	0 (0%) 2 (13%) 2 (13%) 4 (26%) 6 (40%) 1 (7%) 9 (60%) 1 (7%) 1 (7%)	16 (80%) 13 (65%) 12 (60%) 1 (0%) 2 (10%) 5 (25%) 13 (65%) 2 (10%) 5 (25%)	– – – – – – – – –
Lesion location Brain Spinal cord Brian and spinal cord	8 (8%) 81 (79%) 9 (8%)	15 (100%) 4 (26%) 4 (26%)	16 (80%) 4 (20%) 2 (10%)	– – –
CSF WBC × 10^6^ (median (minimum‐maximum))	0 (0–420)	24 (10–67)	28.75 (8–96)	0 (0–3)
CSF YKL‐40 (pg/ml, mean ± SD)	210.52 ± 161.62	93.56 ± 40.91	129.11 ± 46.03	63.18 ± 9.22
CSF sCD14 (pg/ml, mean ± SD)	87.23 ± 56.85	71.66 ± 26.20	85.16 ± 38.24	58.14 ± 7.66
CSF sCD163 (pg/ml, mean ± SD)	68.22 ± 24.11	79.04 ± 9.70	75.97 ± 16.54	55.76 ± 9.48
EDSS scores (median (minimum‐maximum))	3 (0,9)	2 (1,3)	3 (1,5.5)	–
Course (day, interquartile range)	12 (7,30)	2 (3,4.5)	10 (7,15)	–

Abbreviations: CSF, cerebrospinal fluid; NMOSD, neuromyelitis optica spectrum disorders; RRMS, remitting relapsing multiple sclerosis; CTLs, controls; WBC, white blood cell.

### 2.2 Determination of CSF levels of YKL‐40, sCD14, and sCD163

After the patients were admitted, they underwent lumbar puncture for CSF analysis before treatment. The CSF samples were centrifuged immediately after collection to separate the cells and larger particles and were then stored at −80°C until the assays were performed. Commercial sandwich enzyme‐linked immunosorbent assay (ELISA) kits were used to detect the CSF concentrations of YKL‐40 (Quantikine ELISA, R&D Systems, Minneapolis, MN, USA), sCD14, and sCD163 (Bender Med Systems GmbH, Vienna, Austria). ELISA assays were performed in accordance with the manufacturers’ instructions.

### 2.3 EDSS scores

Two researchers determined the EDSS scores of all enrolled patients at the initial stage of admission.

### 2.4 Statistical analysis

SPSS 20.0 (IBM Corp., Armonk, NY, USA) was used for the statistical analysis, and data are expressed as the mean ± standard deviation. The Kruskal–Wallis test was used to analyze the differences in YKL‐40, sCD14, and sCD163 levels among the subgroups. The Pearson's test or Spearman's test was used to evaluate correlations between YKL‐40, sCD14, and sCD163 levels and EDSS scores. A value of *p* < .05 was considered statistically significant.

## 3 RESULTS

### 3.1 Demographic and clinical characteristics of patients

As shown in Table 1, there were no significant differences in age or sex among the groups. The EDSS scores and CSF white blood cell counts were not significantly different between patients in the NMOSD, RRMS, and GFAP groups.

### 3.2 CSF YKL‐40, sCD14, and sCD163 levels

**FIGURE 1:**
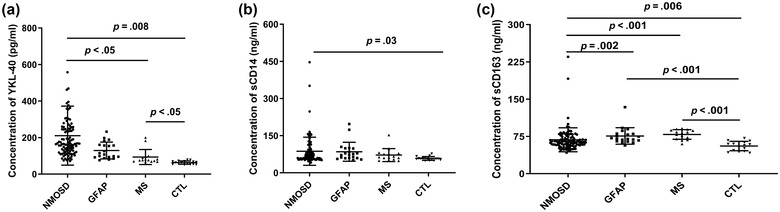
The cerebrospinal fluid (CSF) levels of YKL‐40, sCD14, and sCD163 are shown. (a) The CSF YKL‐40 concentration in patients with neuromyelitis optica spectrum disorder (NMOSD) was significantly higher than those in the relapsing‐remitting MS ((RRMS) and control groups (*p* < .05; *p* = .006). The CSF YKL‐40 concentration in the MS group was also significantly higher than that in the control group (*p* < .05). (b) The CSF sCD14 concentration in patients with NMOSD was significantly higher than that in the control group (*p* = .03). (c) The CSF sCD14 concentration in patients with NMOSD was significantly higher than those in the glial fibrillary acidic protein (GFAP), RRMS, and control groups (*p* = .002; *p* < .001; *p* = .006); the CSF sCD14 concentrations in the GFAP and RRMS groups were significantly higher than that in the control group (*p* < .001; *p* < .001).

As shown in Figure 1, YKL‐40, sCD14, and sCD163 concentrations were detected in CSF samples from the NMOSD (*n* = 103), RRMS (*n* = 15), GFAP (*n* = 20), and control (*n* = 16) groups. The mean concentrations of YKL‐40 (pg/mL) were 210.52 ± 161.62 for the NMOSD group, 93.56 ± 40.91 for the RRMS group, 129.11 ± 46.03 for the GFAP group, and 63.18 ± 9.22 for the control group. The YKL‐40 concentrations in the NMOSD group were significantly higher than those in the RRMS (*p* < .005) and control (*p* < .005, Figure 1a) groups, but there was no significant difference between the NMOSD and GFAP groups. The YKL‐40 content in the RRMS group also exceeded that in the control group (*p* < .05). The levels of sCD14 and sCD163 in the NMOSD group were significantly higher than those in the control group (*p* < .05, *p* < .05, Figure 1b,c). Additionally, there were significantly higher levels of sCD163 in the NMOSD group than those in the GFAP and RRMS groups (*p* < .05, *p* < .001, Figure 1b,c), as well as between the control group and the GFAP and RRMS groups (*p* < .001, *p* < .001). When sCD14 levels were compared among the inflammatory demyelinating disease subgroups, there were no significant differences within these groups or compared with the control group.

### 3.3 Receiver operating characteristic curve analysis

**FIGURE 2:**
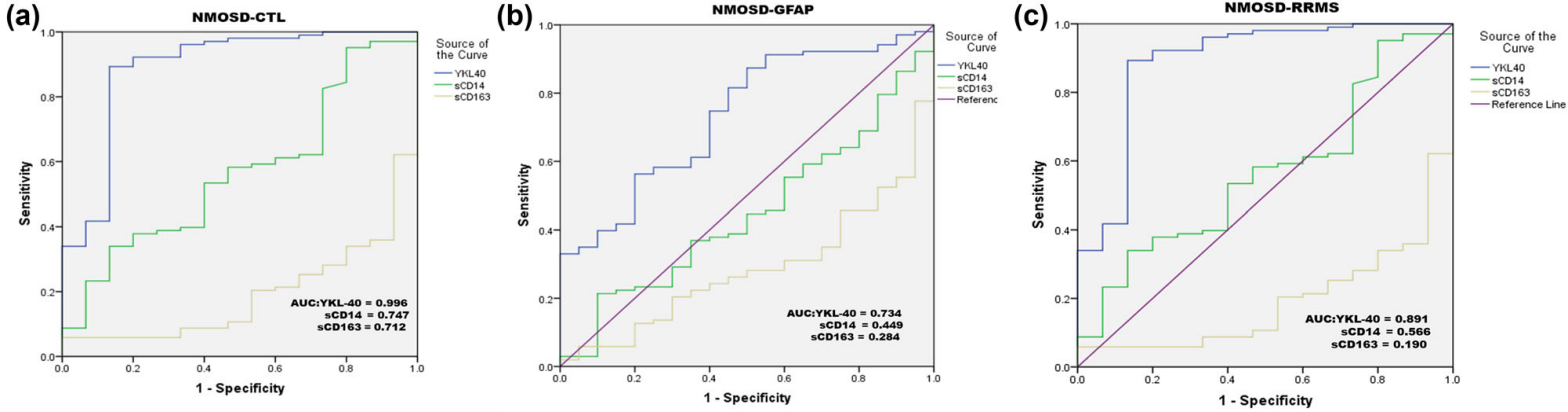
The receiver operating characteristic (ROC) curve analysis of cerebrospinal fluid (CSF) YKL‐40, sCD14, and sCD163 in patients with neuromyelitis optica spectrum disorder (NMOSD) to distinguish from CTL patients (a), patients with relapsing–remitting multiple sclerosis (RRMS) (b), and patients with glial fibrillary acidic protein (GFAP) (c)

We performed receiver operating characteristic (ROC) analysis for YKL‐40, sCD14, and sCD163 to distinguish patients with NMOSD from GFAP/RRMS/control (CTL) patients in Figure 2. The area under curve (AUC) for YKL‐40 in patients with NMOSD to distinguish from CTL patients was 0.996 (95% CI: 0.988–1.000, *p* = .000), the AUC for sCD14 was 0.747 (95% CI: 0.644–0.850, *p* = .002), the AUC for sCD163 was 0.712 (95% CI: 0.582–0.843) (Figure 2a). The AUC for YKL‐40 in patients with NMOSD to distinguish from RRMS patients was 0.891 (95% CI: 0.784–0.997, *p* = .000), the AUC for sCD14 was 0.566 (95% CI: 0.419–0.713, *p* = .408), and the AUC for sCD163 was 0.190 (95% CI: 0.094–0.285, *p* = .000) (Figure 2b). The AUC for YKL‐40 in patients with NMOSD to distinguish from GFAP patients was 0.734 (95% CI: 0.622–0.846, *p* = .001), the AUC for sCD14 was 0.449 (95% CI: 0.321–0.576, *p* = .065), and the AUC for sCD163 was 0.284 (95% CI: 0.181–0.388, *p* = .053) (Figure 2c).

### 3.4 Correlations among YKL‐40, sCD14, and sCD163

**FIGURE 3:**
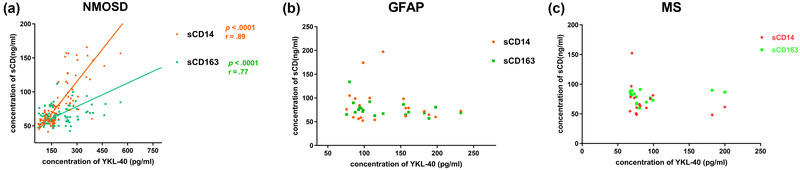
Correlations between YKL‐40, sCD14, and sCD163 are shown. YKL‐40 concentrations in cerebrospinal fluid (CSF) were positively correlated with sCD14 and sCD163 in patients with neuromyelitis optica (NMO) (sCD14, *p* < .001; sCD163, *p* < .001). sCD14 concentrations in CSF were positively correlated with sCD163 in patients with NMO (*p* < .001).

Because the concentrations of YKL‐40, sCD14, and sCD163 were all increased in the NMOSD, GFAP, and RRMS groups, we examined the correlations among these three markers. In the NMOSD group, YKL‐40 levels were significantly correlated with sCD14 (*r* = 0.89, *p* < .0001) and sCD163 (*r* = 0.77, *p* < .0001) levels, and sCD14 levels were positively correlated with sCD163 levels (*r* = 0.77, *p* < .0001). However, there were no correlations among YKL‐40, sCD14, and sCD163 in the GFAP or RRMS groups (Figure 3a,b).

### 3.5 Correlations between CSF profiles and EDSS scores

**FIGURE 4:**
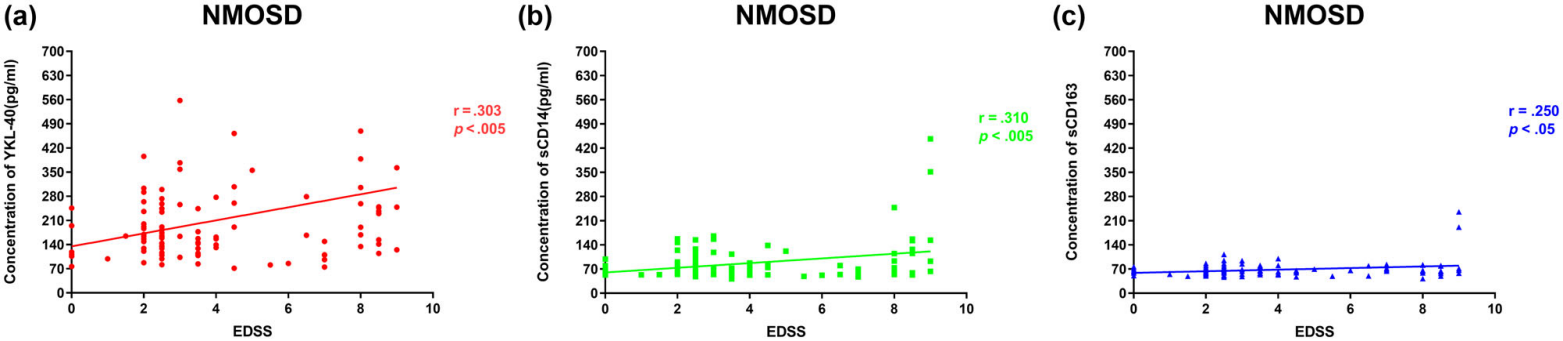
Associations between cytokines in the cerebrospinal fluid (CSF) and the Expanded Disability Status Scale (EDSS) score are shown in patients with neuromyelitis optica. CSF levels of YKL‐40 (a), sCD14 (b), and sCD163 (c) were associated with EDSS scores (*p* < .005; *p* < .005; *p* < .05).

**FIGURE 5:**
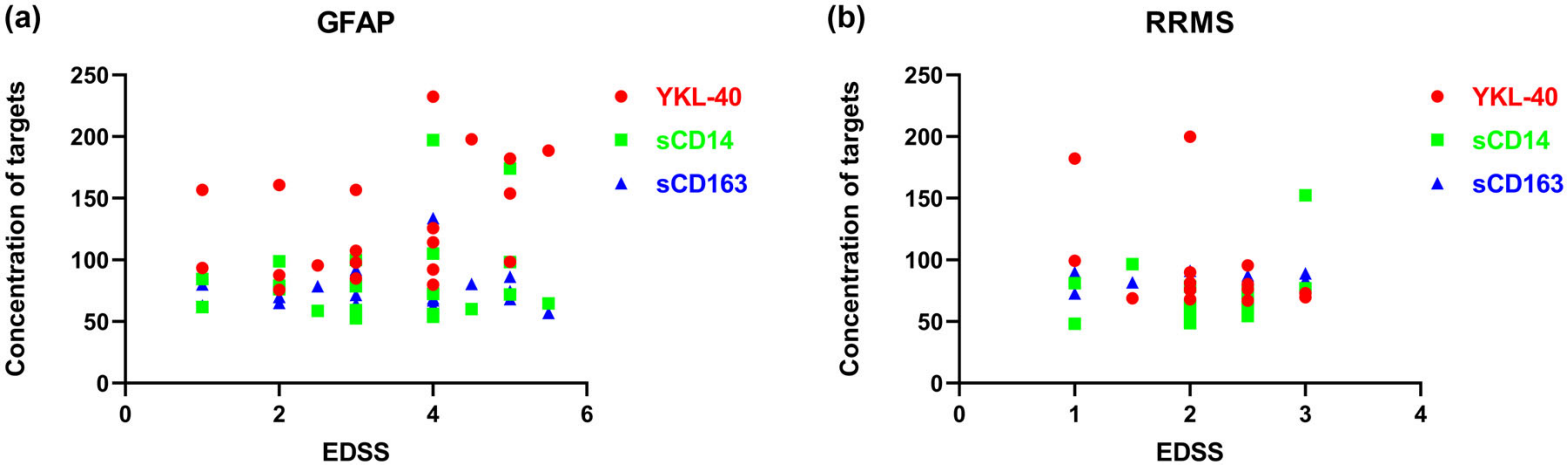
Associations between cytokines in the cerebrospinal fluid (CSF) and the Expanded Disability Status Scale (EDSS) score are shown in relapsing–remitting multiple sclerosis (RRMS) and glial fibrillary acidic protein (GFAP) groups. Cytokines in RRMS groups (a) and GFAP groups (b) had no significant correlation with EDSS scores.

As shown in Figure 2a–c, in patients with NMOSD, disease severity (EDSS scores) was higher in patients with elevated levels of YKL‐40, sCD14, and sCD163 (*p* < .05). (Figure 4a–c). However, the CSF YKL‐40, sCD14, and sCD163 levels were not associated with EDSS scores in the RRMS or GFAP groups (Figure 5a,b).

## 4 DISCUSSION

In this case–control study, we confirmed that the CSF concentrations of YKL‐40, sCD14, and sCD163 were increased in patients with NMOSD. Moreover, these biomarkers were all significantly associated with disease severity. This is consistent with the results published by Qi et al. (2020). However, they only investigated the correlation between YKL‐40 and NMOSD, whereas in our study, the relationship between YKL‐40, sCD163, and sCD14 was investigated simultaneously. To the best of our knowledge, this study is the first to report changes in YKL‐40, sCD14, and sCD163 levels in the CSF of patients with NMOSD.

NMOSD is an autoimmune disease characterized by optic neuritis and myelitis. The exact mechanisms of neuronal damage in NMOSD remain unclear. However, recently increasing evidence has demonstrated that highly reactive microglia/macrophages are a key feature of NMOSD (Guo et al., 2017; B. F. Popescu & Kao, 2011; A. Popescu & Lucchinetti, 2012; Saji et al., 2013). The conspicuous activation of microglia/macrophage has been observed and is considered to have a pathogenic role in actively demyelinating spinal cord lesions of patients with NMOSD (Lucchinetti et al., 2014; Stilund et al., 2014). It is currently understood that microglia/macrophages not only have a simple reaction to injury, infection, or a pathological state, but can also repair CNS structure, refine the regulation of neural circuit and network connectivity, and play a role in the formation of nervous system plasticity (Salter & Beggs, 2014). However, it remains to be further clarified whether microglial/macrophage activation is beneficial or harmful. Microglial/macrophage activation can be revealed by sCD14, sCD163, and YKL‐40 expression; these biomarkers have well‐known implications in brain diseases, such as MS, intracranial infections, and neurodegenerative diseases (Bonneh‐Barkay et al., 2010; Dheen et al., 2007), However, little is known about these biomarkers in NMOSD.

Our data contribute to the growing recognition of the role of microglia/macrophages in demyelinating neuropathies such as NMOSD. In our study, the elevated levels of microglial/macrophage activation markers in the CSF of patients with NMOSD were positively correlated with EDSS scores, further indicating that microglia/macrophage activity is correlated with disease severity. These findings suggest that microglial/macrophage activation, as revealed by YKL‐40, sCD14, and sCD163 concentrations, can be used to assess the severity of NMOSD and may serve as markers to monitor the effects of treatment and predict disease prognosis.

Our research has several limitations. First, with the exception of the NMOSD group, the sample sizes of the other study groups were relatively small, so we need to be cautious when interpreting the results of these groups. Second, the biomarkers investigated in this study are not specific to microglia/macrophages and may involve other inflammatory cells, which may affect the value of our conclusions. Finally, this was only a preliminary study, and the mechanisms of microglial/macrophage activation in NMOSD remain unclear and should be clarified in our further studies.

## 5 CONCLUSIONS

We revealed increased sCD14, sCD163, and YKL‐40 levels in the CSF of patients with NMOSD. The levels of these markers were positively correlated with EDSS scores in these patients, suggesting that microglial/macrophage activation is involved in the pathogenesis of NMOSD. To fully understand the role of microglial/macrophage activation in NMOSD, additional studies are needed.

## CONFLICT OF INTEREST

The authors declare no conflict of interest.

### PEER REVIEW

The peer review history for this article is available at https://publons.com/publon/10.1002/brb3.2798

## Data Availability

The raw data are available by email on reasonable request to the corresponding author.
